# Malnutrition in adult patients treated with venoarterial extracorporeal membrane oxygenation: A descriptive cohort study

**DOI:** 10.1016/j.xjon.2024.09.029

**Published:** 2024-10-16

**Authors:** Stacy Pelekhaty, Julie Gessler, Devon Baer, Raymond Rector, Michael Plazak, Allison Bathula, Chris Wells, Aakash Shah, Alison Grazioli, Bradley Taylor, Bartley P. Griffith, Joseph Rabin

**Affiliations:** a Department of Clinical Nutrition, University of Maryland Medical Center, Baltimore, Md; b Department Perioperative Services, University of Maryland Medical Center, Baltimore, Md; c Department of Pharmaceutical Services, University of Maryland Medical Center, Baltimore, Md; d Department of Rehabilitation Services, University of Maryland Medical Center, Baltimore, Md; e Department of Cardiothoracic Surgery, University of Maryland Medical Center, Baltimore, Md; f Department of Medicine, University of Maryland School of Medicine, Baltimore, Md; g Division of Cardiac Surgery, University of Maryland School of Medicine, Baltimore, Md; h Department of Surgery, University of Maryland School of Medicine, Baltimore, Md

**Keywords:** VA ECMO, malnutrition, nutrition

## Abstract

**Objective:**

To evaluate malnutrition and its association with outcomes in adult patients requiring venoarterial (VA) extracorporeal membrane oxygenation (ECMO).

**Methods:**

Patients cannulated for VA ECMO between January 1, 2020, and January 1, 2023, were screened. Patients on ECMO for <48 hours or without a nutritional evaluation were excluded. Demographic and anthropometric data were collected retrospectively. Malnutrition assessments were conducted using the Global Leadership Initiative on Malnutrition framework. Outcomes analyzed were duration of ECMO and in-hospital mortality. Patients were stratified by admission and discharge nutritional status for analysis. Baseline characteristics were controlled for with propensity score matching.

**Results:**

Data from 197 patients was analyzed. The cohort was 68% male. The median duration of ECMO was 139.5 hours (interquartile range [IQR], 94.8-257 hours), and mortality was 35%. Thirty-three patients presented with malnutrition, and 61 developed hospital-acquired malnutrition, for an incidence of 47.7%. Malnutrition at any point was associated with longer duration of ECMO (median, 180 hours [IQR, 107.8-335.8 hours] vs 120 hours [IQR, 90-185.8 hours]; *P* < .001). Patients with hospital-acquired malnutrition required a 50% longer duration of ECMO (median, 182.5 hours [IQR, 101.5-367 hours] vs 123 hours [IQR, 90.8-211.5 hours]; *P* < .001). Preexisting malnutrition was associated with a nonsignificant increase in mortality (48.2% vs 32.9%; *P* = .13), which was similar after 3:1 propensity score matching (43.3% vs 35.4%; *P* = .44).

**Conclusions:**

In adult patients, malnutrition appears to be associated with prolonged duration of VA ECMO. Adequately powered studies are needed to further investigate the relationship between malnutrition and mortality.

## Graphical Abstract

**Figure:**
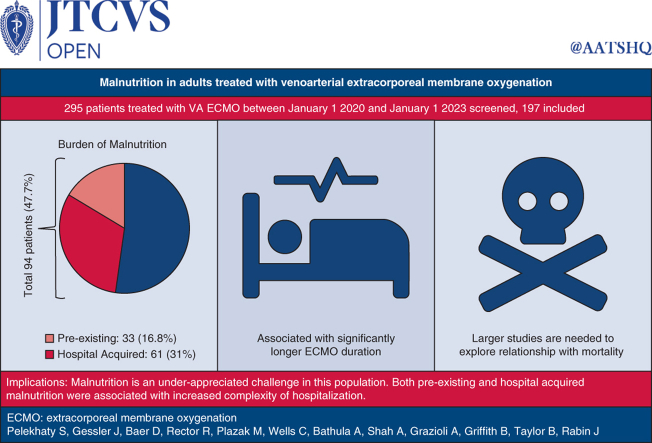

**Figure:**
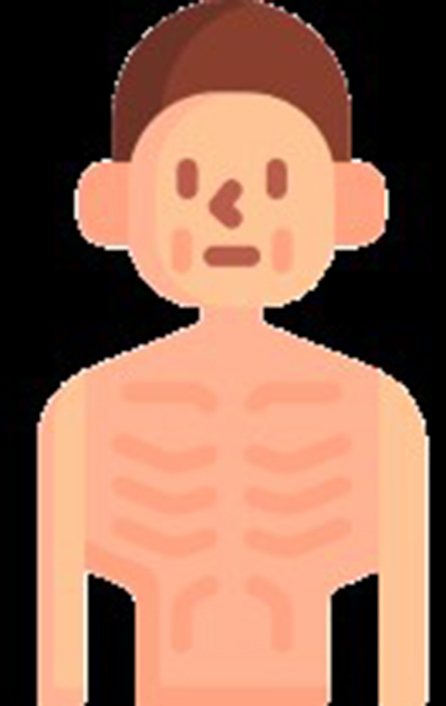
Malnutrition often is identified by muscle depletion on physical exam (source: https://www.freepik.com/icon/malnutrition_4244626).

**Central Message**

Hospital-acquired and preexisting malnutrition are associated with increased duration of venoarterial extracorporeal membrane oxygenation. The relationship between mortality and malnutrition in this population requires further investigation.

**Perspective**

Awareness of the nutritional status of patients on venoarterial extracorporeal membrane oxygenation (ECMO) prior to hospitalization and during the course of ECMO is an important component of holistic patient care. Poor nutritional status may complicate the care of ECMO patients, as it is associated with increased ECMO duration. The relationship between mortality and preexisting malnutrition requires additional study.

See Discussion on page 47.

Malnutrition diagnosed at any stage during hospitalization is associated with increased rates of adverse clinical outcomes. A 2021 study by Guenter and colleagues^1^ found that hospitalized adults diagnosed with malnutrition or a malnutrition-related condition experienced longer hospital stays, higher mortality rates, and lower likelihood for survivors to be discharged to home.^1^ Malnutrition also is associated with increased rates of hospitalization among adult patients presenting to the emergency department and with increased health care costs regardless of admission status.^2^ In the United States, malnutrition is diagnosed in only 3.6% of cases presenting to the emergency department and in 8.9% of hospitalized adults according to retrospective database analyses^1^^,^^2^; however, the true prevalence of malnutrition on arrival is estimated to be at least 33% of hospitalized patients.^3^ Despite malnutrition's known deleterious effects, it continues to be an underrecognized and underdiagnosed factor affecting the clinical course of hospitalized adults.

Adult patients supported with extracorporeal membrane oxygenation (ECMO) are typically at high risk of having or developing malnutrition during hospitalization, related to the complexity of their care and severity of their critical illness.^4^ This is substantiated by multiple retrospective studies reporting that adults undergoing ECMO frequently do not meet nutritional targets and that VA ECMO support may be associated with lower rates of enteral nutritional adequacy compared to venovenous (VV) ECMO.^5^, ^6^, ^7^, ^8^, ^9^ Additionally, Zhu and colleagues^10^ found that being deemed at high risk of developing malnutrition according to validated screening tools was independently associated with increased mortality in adult ECMO recipients. In addition, barriers to receiving adequate nutritional support while in the intensive care unit further increase the risk of developing malnutrition and may be more pronounced in patients on VA ECMO compared to VV ECMO.^4^ Such barriers to nutritional adequacy include the need for high-dose vasoactive support, gastrointestinal intolerance to enteral nutrition (EN), and frequent interruption of EN for operations and invasive procedures.^4^ Additionally, parenteral nutrition (PN) may be avoided because of the historically increased risk for infectious complications, concern for lipid emulsions interfering with oxygenator durability or function, and fluid restrictions.^4^

Although it has been documented that patients on VA ECMO are at high nutritional risk and often experience suboptimal nutritional support, the prevalence and effects of malnutrition in this population are poorly understood. This retrospective, observational study aimed to evaluate the frequency of malnutrition in adults supported with VA ECMO at a high-volume ECMO center, and to investigate the associations between a diagnosis of malnutrition and clinical outcomes, including duration of ECMO support and mortality.

## Methods

Consecutive patients who received VA ECMO support at a single center in an adult intensive care unit between January 1, 2020, and January 1, 2023, were identified from institutional records and screened for this study. Patients were excluded from the analysis if they received ECMO for <48 hours at this institution, were not assessed by a Registered Dietitian (RD), or received >7 days of VA ECMO at another institution prior to admission. For patients with more than one ECMO run during the data collection period, only the first course of ECMO was included. Medical records were reviewed, and data were abstracted. This research was approved by the center's Institutional Review Board on July 10, 2023 (approval HP-00106249).

### Baseline Data

Demographic data, including age, sex, and primary indication for ECMO, were obtained from the electronic medical record. The RD's note was reviewed for anthropometric measures, including height (cm), weight (kg) at admission and discharge, and admission body mass index (BMI). Survival After Veno-Arterial ECMO (SAVE) scores were calculated for all included patients.^11^

### Malnutrition Assessment

Assessments of nutritional status were performed using the Global Leadership Initiative on Malnutrition (GLIM) consensus criteria.^12^ The GLIM framework includes a 2-step diagnostic process, with patients first assessed for malnutrition risk using any validated screening tool and then comprehensive nutrition assessment completed to determine the presence of malnutrition.^12^ To be diagnosed with malnutrition, a patient must have both an etiologic criteria or cause of malnutrition as well as a temporally related phenotypic criterion or physical manifestation of malnutrition. Under GLIM, the 2 etiologic criteria are either reduced nutritional intake or assimilation or inflammation. Assimilation refers to the absorption, metabolism, and physiologic utilization of nutrients through normal. Reduced nutritional intake may refer to starvation, poor oral intake from gastrointestinal symptoms, or inadequate delivery of nutritional support. Inflammation may be chronic or acute in nature. The framework includes 3 phenotypic criteria: low BMI, weight loss >10% of usual body weight, and low muscle mass. The significance of the phenotypic criteria is used to determine the severity of malnutrition according to the framework cutoffs.

Given that patients on VA ECMO can be anticipated to require admission to an intensive care unit for >48 hours, they should be considered at high risk for malnutrition according to the European Society for Parenteral and Enteral Nutrition guidelines.^13^ The electronic medical record was reviewed to identify both a documented diagnosis of malnutrition by the RD as well as documented phenotypic and etiologic criteria that would qualify a patient as malnourished under the GLIM framework. This RD documentation, including etiology of malnutrition, weight history, BMI, and muscle wasting as assessed by a nutrition-focused physical exam (NFPE) was abstracted. Additionally, the severity of malnutrition, as defined by the phenotypic criteria according to the GLIM framework, was recorded. Nutritional status was recorded at the time of cannulation for VA ECMO and at the time of hospital discharge. Preexisting malnutrition was defined as malnutrition identified in the first RD assessment after cannulation, and malnutrition present at the time of discharge in a patient without preexisting malnutrition was considered hospital-acquired.

### Nutrition Protocol

All patients are evaluated by an RD within 3 days of cannulation. There currently are no published recommendations for energy or protein targets specific to patients on ECMO; therefore, our institution developed a nutrition protocol guided by population-specific literature to best address the needs of this unique subset of critically ill adults. In patients with a BMI <30, energy needs are assessed as 18 to 22 kcal/kg/day, and protein needs are assessed as 2 to 2.5 g/kg/day using actual body weight.^14^^,^^15^ For patients with a BMI of ≥30, energy targets are set at 20 to 25 kcal/kg/day, and protein needs are estimated as ≥2.5 g/kg/day using ideal body weight calculated according to the method of Hamwi.^14^, ^15^, ^16^ According to institutional guidelines, energy needs can be assessed using the Penn State equations, with minute ventilation of 0 L/minute as a cross-reference to the aforementioned kcal/kg targets.^17^^,^^18^ In patients on ECMO who do not require mechanical ventilation, nutritional needs are met through oral diets. The RD assesses the adequacy of oral intake and prescribes supplementation as indicated. In patients unable to meet their nutritional needs through oral intake owing to either poor appetite or mechanical ventilation, nutritional support is preferentially provided enterally, in accordance with published guidelines.^19^ PN is recommended when nutritional adequacy cannot be achieved via oral or enteral routes.^19^^,^^20^ Nutritional adequacy is defined as meeting at least 55% to 65% of nutritional targets over the first 7 to 10 days of critical care and at least 80% of nutritional targets thereafter.^19^

### Analysis

The primary outcomes of interest were survival to discharge and the duration of ECMO. An a priori power analysis to detect a 20% difference in in-hospital mortality between malnourished and well-nourished patients calculated a minimum of 96 patients per group, assuming that mortality in the malnourished patients was 50%, or roughly equivalent to ELSO statistics for cardiac ECMO.^21^ A 20% in-hospital mortality difference was estimated based on prior nutritional studies that demonstrated a 15% to 30% increase in mortality among malnourished patients.^22^ This center manages approximately 100 patients on VA ECMO annually, and the data collection period was set at 3 years to allow for screening of 300 patients for study inclusion.

Baseline data were summarized for the full cohort, and overall rates of malnutrition were calculated. Patients were then stratified based on whether malnutrition was present at any point during hospitalization and clinical outcomes were compared. Subanalysis was conducted after patients were stratified by admission nutritional status. To assess the impact of hospital-acquired malnutrition, patients without malnutrition on arrival who subsequently developed malnutrition were compared to patients who did not develop malnutrition. Malnourished patients may have reduced nutritional stores and thus may be less able to withstand prolonged critical illness; therefore, we conducted a subanalysis among patients who required ≥5 days of VA ECMO. Data were summarized as median (interquartile range [IQR]), mean ± standard deviation (SD), or count (frequency), as appropriate. Between-group comparisons were made with the Student *t*, Mann-Whitney U, χ^2^, or Fisher exact test, as indicated.

To account for differences in baseline covariates between patients with and without preexisting malnutrition, propensity score matching was performed. First, a multivariable logistic regression was used to predict the probability of preexisting malnutrition. The covariates of age, sex, BMI, and SAVE score were entered into the final model. Patients with no preexisting malnutrition and those with preexisting malnutrition were matched 3:1 via a nearest-neighbor matching algorithm without replacement. A caliper of 0.2 of the standard deviation of the propensity score on the logit scale was selected.^23^ Covariate balance between the 2 groups was assessed after matching. An absolute standardized difference <0.1 was considered evidence of balance. Finally, outcomes were compared with an unadjusted analysis between the preexisting malnutrition and no preexisting malnutrition groups within the matched dataset.^24^ All propensity score matching was performed using the *MatchIt* package with R version 4.3.1 (R Foundation for Statistical Computing).

## Results

**Figure 1:**
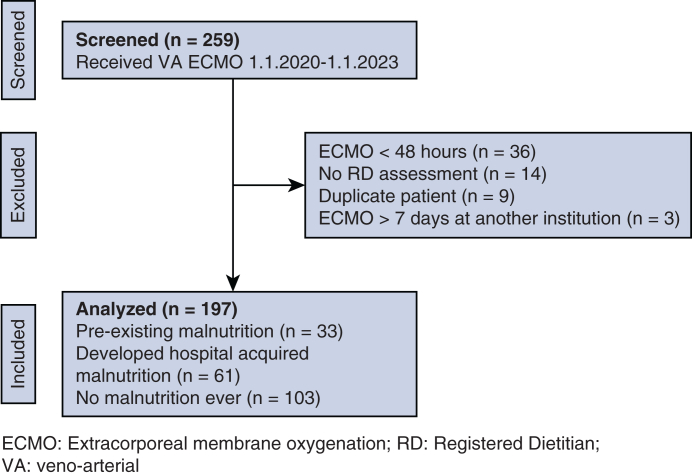
A flow diagram of patients screened for inclusion, reasons for exclusion, those which are included in the final analysis for this research. *VA*, Venoarterial; *ECMO*, extracorporeal membrane oxygenation; *RD*, Registered Dietitian.

A total of 259 patients were screened, and 197 patients were included in our analysis. Sixty-two patients were excluded for inadequate duration (<48 hours) of ECMO support (n = 36), RD assessment not available (n = 14), patient included for a previous ECMO run (n = 9), and prolonged cannulation at another institution (n = 3) (Figure 1). Baseline characteristics and primary indication for VA ECMO are summarized in Table 1 for the full cohort and after stratification for admission nutrition status. Overall, the cohort was 68% male and had a median age of 56 years (IQR, 45-63 years) and a median BMI of 29.9, consistent with overweight. Patients who presented with malnutrition weighed less and had a significantly lower BMI compared to those without malnutrition (Table 1).

**Table 1:** Baseline characteristics of adult VA ECMO patients overall and after stratification for baseline malnutrition status

Characteristic	All (N = 197)	No preexisting malnutrition (N = 164)	Preexisting malnutrition (N = 33)	*P* value
Male sex, n (%)	134 (68)	108 (65.9)	26 (78.8)	.16
Age, y, median (IQR)	56 (45-63)	55 (44-63)	57 (47-63)	.53
BMI, kg/m^2^, median (IQR)	29.9 (25.3-34.1)	30.4 (26.2-34.8)	23.7 (21.3-30)	<.001
Weight, kg, median (IQR)				
Admission	89.8 (72.7-104.3)	90.9 (78.1, 107.5)	69 (63-96.5)	<.001
Discharge	85 (69.6-102.1)	86.8 (75.4, 104.8)	65 (62-84.7)	<.001
Change during hospitalization	0 (−5.4 to 0)	0 (−5.6 to 0)	−0.7 (−5.1 to 0)	.18
SAVE score, mean ± SD	−6 ± 5.8	−5.9 ± 5.7	−6.4 ± 6.5	.65
Indication for ECMO, n (%)				.065
Cardiomyopathy/heart failure	14 (7.1)	9 (5.5)	5 (15.2)	
Cardiac arrest	44 (22.3)	39 (23.8)	5 (15.2)	
Cardiogenic shock	62 (61.5)	56 (34.2)	6 (18.2)	
Postoperative	19 (9.6)	15 (9.2)	4 (12)	
Hypothermia	3 (1.5)	1 (0.6)	2 (6)	
Post-transplant	7 (3.6)	6 (3.7)	1 (3)	
Pulmonary embolism	18 (9.1)	15 (9.2)	3 (9.1)	
Other	30 (15.2)	23 (14)	7 (21.2)	

*VA*, Venoarterial; *ECMO*, extracorporeal membrane oxygenation; *IQR*, interquartile range; *BMI*, body mass index; *SAVE*, Survival After Venoarterial ECMO.

### Nutritional Interventions

Nutritional interventions are summarized in Table 2. The preferred method for nutritional support in this cohort was via the enteral route, with 76% of patients receiving EN. EN was initiated on a median of postcannulation day 2 (IQR, day 1-4), although timing varied widely. PN support was used less frequently and was started later after cannulation (*P* < .001). Additionally, 92.4% of patients (n = 182) advanced to an oral diet either while on ECMO support or after decannulation.

**Table 2:** Nutritional interventions in patients on VA ECMO

Route	PN	EN
Patients, n (%)	27 (13.7)	150 (76.1)
Timing of initiation, d		
Median (IQR)	5 (2-9)	2 (1-4)
Minimum-maximum	0-41	0-65

*VA*, Venoarterial; *EMCO*, extracorporeal membrane oxygenation; *PN*, parenteral nutrition; *EN*, enteral nutritition; *IQR*, interquartile range.

### Incidence of Malnutrition

Twenty-seven patients (13.7%) had an RD diagnosis of malnutrition at the time of cannulation for VA ECMO. Another 6 patients had criteria qualifying them as malnourished on chart review, for a total incidence of 16.8% (n = 33). Seven of these patients presented with severe malnutrition, and the remaining 26 were found to be moderately malnourished. The most common phenotypic criterion was muscle wasting (n = 23), followed by weight loss (n = 13) and low BMI (n = 4). More than 1 phenotypic criterion was observed in 8 patients, which did not correspond to the severity of malnutrition. Severe malnutrition was justified by >10% weight loss in 2 patients and moderate to severe muscle mass depletion in the remaining 5 patients. Nutritional status declined in 74 patients (37.6%), including 5 patients with weight loss that did not meet the diagnostic threshold for malnutrition, 61 initially well-nourished patients who developed hospital-acquired malnutrition, and 8 patients admitted with moderate malnutrition that progressed to severe malnutrition during hospitalization. A total of 94 patients (47.7%) met the criteria for malnutrition while hospitalized.

### Mortality

Among the full cohort, 40 patients (20.3%) died while on ECMO and 29 decannulated patients died prior to discharge, for a total in-hospital mortality of 35% (n = 35). There was a non–statistically significant higher rate of in-hospital mortality in patients who presented with malnutrition compared to those who did not (48.1% vs 32.9%; *P* = .09). For patients who presented without malnutrition, there was no significant difference in mortality between those who developed malnutrition and those who did not (24.4% vs 36%; *P* = .2).

A total of 117 patients required VA ECMO for ≥5 days. Malnutrition was diagnosed by the RD in 16 patients who required ECMO for at least 5 days, and an additional 4 patients had criteria qualifying them as malnourished without a formal diagnosis, for a total of 20. ECMO mortality was higher in the 16 patients diagnosed with malnutrition by an RD compared to patients not diagnosed with malnutrition (37.5% vs 17.8%; *P* < .001). In-hospital mortality also was higher in patients with a diagnosis of malnutrition; however, the difference was not statistically significant (63.5% vs 37.6%; *P* = .1). When considering all patients meeting the criteria for malnutrition, nutritional status at the time of cannulation was associated with a non–statistically significant increase in mortality while on ECMO (30% for malnourished vs 18.6% for not malnourished; *P* = .24) and throughout the entire hospitalization (50% for malnourished vs 39% for not malnourished; *P* = .46).

### Duration of VA ECMO

Overall, the median duration of VA ECMO was 139.5 hours (IQR, 94.3-256.3 hours). A diagnosis of malnutrition was associated with a significantly increased duration of ECMO support (median, 180 hours [IQR, 107.8-335.8 hours] vs 120 hours [IQR, 90- 185.8 hours]; *P* < .001). In patients who presented without malnutrition, developing malnutrition was associated with a roughly 50% longer duration of ECMO compared to patients who maintained their nutritional status (median, 182.5 hours [IQR, 101.5-367 hours] vs 123 hours [IQR, 90.8-211.5 hours]; *P* < .001).

### Propensity Score Matching

**Figure 2:**
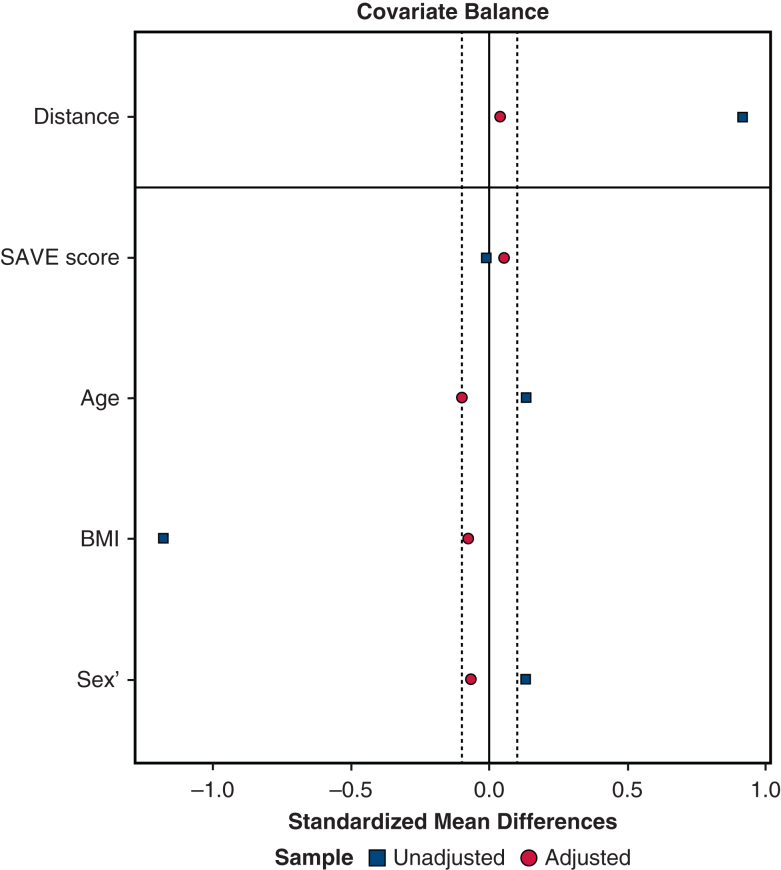
Diagram demonstrating covariate balancing before and after matching. *SAVE*, Survival After Venoarterial ECMO.

To account for differences in baseline characteristics, patients without preexisting malnutrition at ECMO cannulation were matched 3:1 to patients with preexisting malnutrition via propensity score matching. Patients were matched on age, sex, BMI, and SAVE score. Covariate balance before and after matching is depicted in Figure 2. After matching, 30 patients were retained in the preexisting malnutrition group and 82 patients remained in the no preexisting malnutrition group. Among the matched cohort, there was still a non–statistically significantly higher incidence of in-hospital mortality (43.3% for preexisting malnutrition vs 35.4% for no preexisting malnutrition; *P* = .44).

## Discussion

**Figure 3:**
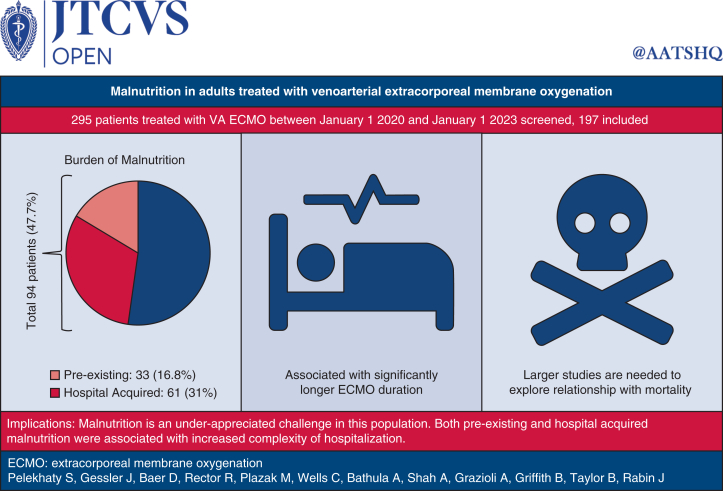
Visual abstract reviewing key methods, results, and clinical implications of the research study. The pie chart visually represents the burden of malnutrition in this population. An additional key finding is the association of malnutrition with longer duration of extracorporeal membrane oxygenation (*ECMO*); however, this study was underpowered to fully explore the relationship between malnutrition and mortality. Clinicians should be aware of the challenges of malnutrition in this population and how a diagnosis of malnutrition may complicate clinical care. *VA*, Venoarterial.

This single-center study is one of the first to investigate the overall incidence and impact of malnutrition in patients supported with VA ECMO (Figure 3). The overall incidence of malnutrition at any time in this cohort of 197 patients was 47.7%, which is similar to published data from general critical illness,^3^ although nutritional status declined in 35% of patients once ECMO support was initiated. The impact of malnutrition on clinical outcomes in critically ill patients has been well documented in the literature.^1^, ^2^, ^3^^,^^22^ Malnutrition in this VA-ECMO cohort was associated with increased complexity of hospitalization. While ECMO duration was increased in patients with malnutrition, it was most pronounced in patients who developed hospital-acquired malnutrition. Finally, although mortality was higher in patients with preexisting malnutrition, the difference did not reach statistical significance. Nonetheless, these results demonstrate that malnutrition is a major underappreciated clinical issue with complex implications in this population and warrants additional attention at the bedside.

These results also should be considered in the context of the excellent overall survival that was achieved, with a 79.7% ECMO survival and 65% survival to discharge. These survival rates surpass the ELSO benchmarks of 61% VA-ECMO survival and 46% survival to discharge.^21^ However, the presence of such a lower mortality rate now requires a larger cohort of patients to identify a significant mortality association.

Multiple methods can be used to diagnose malnutrition, and there is no consensus on what constitutes the optimal strategy or criteria.^25^ Holistic methods have replaced biochemical assessments to diagnose malnutrition, as the use of visceral proteins has been shown to be a poor diagnostic tool, particularly in critically and acutely ill patients.^26^ However, there is no accepted gold standard, and published frameworks have limited validation.^25^ The research presented here used the GLIM framework according to institutional practice standards.^12^ Individual studies have demonstrated that GLIM is an appropriate tool for assessing nutritional status in critically ill adults; however, a recent meta-analysis demonstrated that additional studies are needed to fully validate this method.^27^^,^^28^ This study showed an association between malnutrition assessed using the GLIM framework and clinical outcomes, suggesting that this is a reasonable strategy for diagnosing malnutrition in patients undergoing VA ECMO support.

Beyond diagnostic frameworks, imaging and body composition tools have been implemented in some studies to assess nutritional status. These tools include ultrasound, computed tomography, bioelectrical impedance analysis, and dual X-ray absorptiometry.^29^ Thresholds for the diagnosis of malnutrition require additional validation, and GLIM recommended the use of thresholds validated to identify low muscle mass and sarcopenic conditions, which may or may not indicate malnutrition.^12^ Additionally, tools such as bioelectrical impedance analysis and computed tomography may generate erroneous readings in patients with significant fluid accumulation due to alterations in tissue conductivity or Hounsfield unit measures.^29^^,^^30^ This study used an NFPE to identify muscle wasting and low muscle mass, which is an accepted diagnostic tool.^12^^,^^29^^,^^31^ An NFPE performed routinely at the bedside by trained practitioners was used to support >70% of the malnutrition diagnoses in this cohort. Weight loss was used less frequently as an etiologic criterion, accounting for only 30% of the diagnoses, given the difficulty in distinguishing true weight changes from fluid fluctuations during hospitalization. Additionally, routine weights on reliable scales are difficult to achieve in this population due to their acuity of critical illness. In this population, weight loss may be less impactful on overall outcomes compared to muscle loss, and the validity of specific malnutrition criteria warrants additional investigation. Comparing patients who presented with malnutrition, mortality was higher in the 23 patients who were diagnosed with malnutrition due to muscle wasting compared to the 7 patients who were diagnosed based on weight loss (43.5% vs 28.6%; *P* = .66). Although these differences did not reach statistical significance, the sample sizes were exceptionally small, and type II error cannot be eliminated. Future work investigating malnutrition in adults on VA ECMO should incorporate criteria validation into the study design.

Although prior research has consistently shown that nutrition interventions can improve clinical outcomes in malnourished patients, the question of how nutrition can impact the development or worsening of malnutrition during periods of critical illness remains.^32^, ^33^, ^34^ One recent study in 34 critically ill patients with Coronavirus disease 2019 found that the addition of nutritional supplementation prevented the development of malnutrition^35^; however, nutritional support was required in 90% of the patients in this cohort. In accordance with published guidelines, early enteral support was preferred and initiated on a median of postcannulation day 2, with PN initiated on a median of postcannulation day 5 when early EN failed.^18^^,^^19^ The ability of nutritional interventions to reduce rates of malnutrition in patients requiring ECMO warrants additional research.

The association observed between hospital acquired malnutrition and duration of VA ECMO in this study is likely bi-directional: time on ECMO increases the likelihood of developing malnutrition and malnutrition contributes to likelihood of requiring prolonged ECMO. ECMO is associated with muscle wasting, a key diagnostic criterion for malnutrition under the GLIM framework.^36^ Additionally, administration of nutritional support targets is hampered by procedures, operations, and other clinical considerations, resulting in suboptimal administration.^7^ Finally, increased catabolism during ECMO support has been demonstrated, potentially mediated by increased inflammatory processes.^14^ These factors combined contribute to increasing risk of developing malnutrition during ECMO support. Conversely, muscle wasting and poor nutritional delivery have been associated with poor clinical outcomes.^8^^,^^36^ As a result, patients who survive a prolonged ECMO course likely have a higher rate of malnutrition, and the development of malnutrition in turn further complicates and prolongs the ECMO course.

### Limitations

Although this study contributes to our understanding of the importance of nutrition in optimizing outcomes for ECMO patients, it has several limitations that must be acknowledged. This research was conducted at a single, high-volume ECMO center with specially trained RDs embedded in intensive care units, and thus the results might not be representative of malnutrition rates across all ECMO centers that do not have the same nutritional support practices during ECMO or nutritional resources. Although nutritional intervention type and timing were included, precise quantification of nutritional delivery was not available. While PN typically provides 100% of prescribed targets after initiation, patients receive 40.5% (IQR 24%-52.8%) of EN targets over the first week and 65.5% (IQR, 54.3%-80.5%) over the second week of ECMO support at this institution according to internal quality assurance monitoring. Although this amount of nutritional support is below the target of 80% or more of nutritional prescription cited by published guidelines, this nutritional delivery is still consistent with best achievable international practice.^19^^,^^37^ Furthermore, this cohort included patients managed with oral nutrition alone, which cannot be quantified retrospectively.

Additionally, NFPE rather than imaging was used to assess muscle mass. Although NFPE is an accepted assessment tool, it is subjective and limited in patients with significant subcutaneous fat stores or fluid accumulation. The presence of inflammation was an assumed etiology of malnutrition developed during hospitalization owing to the nature of the population. This was used because common biochemical markers of inflammation are not routinely assessed. While disease process is an accepted proxy for inflammatory metabolism under GLIM, the addition of biochemical measures would have strengthened this assessment.^38^ Details of preexisting malnutrition, such as the duration of the nutritional insult or whether the patient developed malnutrition in a community, congregate living, or hospital setting prior to arrival at this institution, were not collected and might have caused unaccounted-for confounding. Finally, this study failed to meet the power analysis and was underpowered to assess the relationship between nutritional status and mortality.

## Conclusions

Malnutrition, both preexisting and hospital-acquired, is a significant burden in adult patients who require VA ECMO support. The presence of malnutrition may complicate hospitalizations and worsen outcomes in this patient population. Malnutrition was specifically associated with increased duration of ECMO support, whereas the relationship between malnutrition and mortality requires further investigation in a larger cohort of patients.

### Webcast

You can watch a Webcast of this AATS meeting presentation by going to: https://www.aats.org/resources/malnutrition-in-adult-patients-7326.

**Figure:**
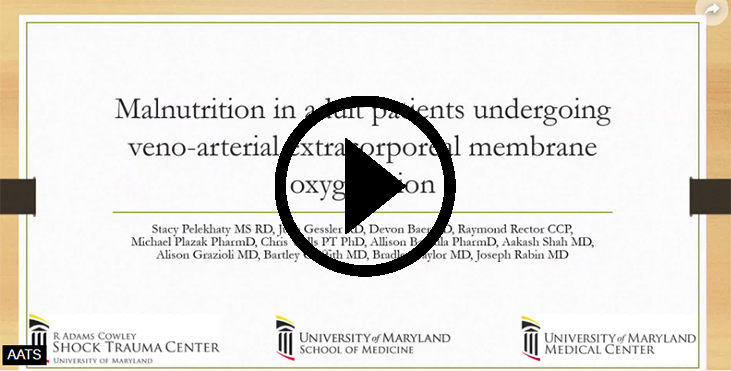

## Conflict of Interest Statement

The authors reported no conflicts of interest.

The *Journal* policy requires editors and reviewers to disclose conflicts of interest and to decline handling or reviewing manuscripts for which they may have a conflict of interest. The editors and reviewers of this article have no conflicts of interest.
